# Nuclear strata: Enacting clay for the deep geological disposal of nuclear waste in Switzerland

**DOI:** 10.1177/25148486251324935

**Published:** 2025-03-13

**Authors:** Rony Emmenegger

**Affiliations:** Department of Geosciences, Switzerland

**Keywords:** Political geology, subterranean, strata, nuclear waste governance, Switzerland

## Abstract

For the realization of a deep geological repository, geoscientists have investigated various geological formations and assessed their suitability as host rocks for the long-term disposal of high-level nuclear waste. A fundamental epistemological uncertainty has characterized this geoscientific exploration of the subterranean, evolving not only as a scientific-technical challenge in deep geological disposal projects but also as a sociopolitical one. This paper scrutinizes how the Swiss nuclear waste organization, during its recent drilling campaign, has publicly staged the Opalinus clay as a stable rock in order to make its disposal project feasible and sound. Based on ethnographic fieldwork as well as document and media analysis, it traces how science communication experts have created and arranged a series of maps, models, and materials to make the subterranean tangible to a surface audience over the course of the campaign. Strategically assembled at specific events, it shows that these political materials did not simply represent subterranean spaces but constituted a stratified subterranean geology on the surface. On this basis, this paper underlines the performative dimension of geology and the key importance of stabilizing geology in science-society encounters for deep geological disposal projects to advance. By illustrating how the subterranean has come to matter and became politically significant in Switzerland's contentious nuclear (waste) history, this paper contributes to a better understanding of the constitutive role of geology for imagining a postnuclear future.

## Introduction

Deep geological disposal is the strategy of safely isolating nuclear waste in a suitable subterranean substrate, removed from the biosphere for up to a million years. This strategy is based upon the concept of passive safety or the idea that the Earth can contain nuclear waste so that active human care is no longer needed (Schröder, 2016). Geology thereby constitutes a key component in a multibarrier system designed as an interplay of natural and engineered barriers, isolating nuclear waste from humans and the environment at the Earth's surface. Clay, crystalline, or salt rock substrates are primarily considered suitable as the so-called host rocks for the final disposal of nuclear waste as they prevent and delay the release of radioactivity to varying degrees through isolation and absorption (Chapman and McCombie, 2003; Sellin and Leupin, 2013). The search for suitable geological formations has turned the subterranean into the focus of geoscience investigation since the 1970s. As science and technology advanced, site exploration provided geoscientists with an increasingly accurate understanding of subterranean conditions necessary for long-term safety evaluation and decision-making.

However, geoscience sensing, drilling, and modeling have not fully resolved the inevitable epistemological uncertainty that characterizes any attempt to understand subterranean spaces from the surface (Murris, 1997)—an uncertainty that increases as deep geological disposal projects require reckoning with deep time for long-term safety evaluation (Emmenegger, 2021; Ialenti, 2020). The epistemic uncertainties characteristic of subterranean exploration are not only a scientific-technical challenge but also a sociopolitical one as they can inspire public controversies—and do so especially when knowledge about the subterranean past and future remains limited to experts (see Kinchy et al., 2018). Mobilized in broader contestations about deep geological disposal, such controversies have been risky as they can undermine geoscience authority and knowledge for political decision-making (see also Stewart and Lewis, 2017). Since the late 1990s, various countries have thus adopted participatory, deliberative or voluntarist governance procedures to ensure public trust and acceptance (e.g., Kuppler, 2012; Lethonen et al., 2022). While this has enabled some success, the realization of deep geological disposal projects has proven to depend not only on sound science and governance but also on a shared belief in the long-term stability of the geological underground (Kearnes and Rickards, 2017).

In fact, the geological disposal concept has been translated into nuclear waste management policies and discourse using rather simplistic terms. For example, Schröder (2016) demonstrates how the international radioactive waste community has promoted it by presuming a steady and stable subterranean geology. Such a conception of the subterranean suggests certainty, rendering deep geological disposal straightforward and sound (see also Schürkmann, 2022). This orientation is part of a strategic “re-imagination of the subsurface” that is paramount for rendering deep geological disposal projects feasible not only in the eyes of experts but also citizens (Kearnes and Rickards, 2017: 55). On a national level, there is evidence that nuclear waste organizations have invested in the discursive framing of geological conditions to foster consent and to convince a critical public about the feasibility of disposal projects (Anshelm and Galis, 2011; Elam and Sundqvist, 2007; Macfarlane, 2003; Sundqvist, 2002). Establishing an epistemic consensus around “a view of nature as fundamentally stable’ (Anshelm and Galis, 2011: 418) appeared particularly significant where understandings of subterranean ontology diverged between experts and citizens (Anshelm and Galis, 2011; Aschwanden, 2020).

This article examines how the Swiss National Co-operative for the Disposal of Radioactive Waste (Nagra) has framed Opalinus clay in public as a stable rock in its latest drilling campaign between 2019 and 2022. As the final stage of a long-term siting process, this campaign has focused on an extensive subterranean Opalinus clay formation—as a favorable host rock for nuclear waste disposal—at three sites in northern Switzerland. As the culmination of a long history of geological exploration (see Hadermann et al., 2014), the campaign was launched to complete the picture of subterranean conditions and provide a basis for selecting the “safest” of the three sites. In addition to its geoscientific relevance, the campaign provided new opportunities for communicating the geoscience involved. Among other activities, Nagra's communication team organized a series of events for media representatives and the uninformed public—all those who were not yet familiar with the topic—to provide information on the ongoing geoscientific investigation and its preliminary results.^1^ In September 2022, the drilling campaign enabled Nagra to publicly announce its assessment of Nördlich Lägern as the safest site—an assertion that has remained relatively uncontested.

This paper examines how Nagra's science communication experts have unearthed the subterranean in public events organized around the drilling. Methodologically, it is based on ethnographic fieldwork between 2019 and 2024 that involved participant observation at various sites of communication and semistructured interviews with communication experts—and that was complemented by document and media analysis. Conceptually, this paper draws on established subterranean scholarship in science and technology studies, political geography, and political geology. This scholarship challenges the assumption of an *a priori* existent subterranean and suggests instead an analysis of the practices and performances through which the subterranean is enacted, or brought into being (Bruun, 2018; Kinchy et al., 2018; Kroepsch, 2018; Marston and Himley, 2021). It follows that the materiality of the subterranean should not be seen as given but as the result of material politics (Barry, 2013)—with materiality being in a “state of becoming” and dependent “on the enactment and stabilization of knowledge claims” (Bakker and Bridge, 2021: 49). Against this background, this paper shows how Nagra's communication experts have publicly enacted a stable Opalinus clay stratum capable of containing nuclear waste for a million years. In particular, it illustrates the importance of maps, models, and materials in making a stratified subterranean tangible to a surface audience. Strategically assembled at specific events, as it shows, these “political materials” (Jensen, 2015) do not simply visualize, represent, or simulate subterranean spaces but constitute subterranean worlds on the surface.

Overall, this paper reflects my ambition to problematize the role of geology in geological disposal projects and to “unearth” (Melo Zurita et al. 2017) the taken-for-granted stability of clay as a host rock. To do so, it accounts for the country's contentious nuclear (waste) history and takes seriously Bosworth's (2023: 13) call for subsurface geographers to “better historicize and explain how and under what conditions an assumption of undynamic inertness comes to be hegemonic.” As such, it provides nuanced insights into the ways in which the Opalinus clay has become politically significant in the search for a deep geological repository in Switzerland. It is structured as follows: section two offers a brief history of the (contested) subterranean exploration in Switzerland since the 1970s and the way it has rendered the subterranean legible to geo-techno-scientific calculations. The following empirical sections of this paper provide insights into Nagra's recent communication, including its materialization in information pavilions at the drill sites; in a public display of the first retrieved drill core; in an exhibition of drill core cross sections representative of the three potential disposal regions; and a national press conference organized for Nagra to justify its site selection. In conversation with existing literature on the subterranean (see Bosworth, 2023), this paper closes with a reflection about how Opalinus clay *came to matter* and became constitutive for imagining Switzerland's postnuclear futures.

## Project opalinus

In line with an international expert consensus, Switzerland prioritizes deep geological disposal as its strategy to safely isolate high-level nuclear waste in the long term (NEA, 2003). For the realization of such a repository, the Swiss program is based upon on Opalinus clay as the favorable host rock. Experts consider the Jurassic argillaceous formation favorable due to its specific rock properties, namely its low permeability, high sorption capacity, and ability to self-seal fractures due to swelling clay particles; disadvantages of the material include limited structural strength and low-thermal conductivity (Sellin and Leupin, 2013). A potentially suitable Opalinus clay formation exists in northern Switzerland as a rather compact formation in a reachable depth of 400–1000m. As this section illustrates, geoscience exploration since the 1970s has not simply described the subterranean in northern Switzerland, but has rendered it legible as an epistemological space, or as a “vertical territory” (Braun 2000), for the geo-techno-scientific calculation and for the advancement of the nuclear waste disposal project.

Opalinus clay gained prominence as a favorable host rock after the initial exploration of crystalline granite formations became scientifically and politically untenable. Providing evidence for the feasibility of deep geological disposal became necessary for the nuclear industry after the 1978 Nuclear Energy Law defined a so-called “proof of disposal” as a prerequisite for any future licensing of nuclear power plants in Switzerland. To that end, Nagra was created already in 1972 with the mandate of finding a technoscientific solution for nuclear waste. It has brought specific host rocks into the focus of scientific investigation—and, increasingly, into the focus of science communication as well. Launching “Project Guarantee” in the early 1980s, Nagra's initial program for high-level waste targeted crystalline formations, granite in particular, in line with similar programs in Sweden and the USA (Milnes et al., 2008). Crystalline rock was considered suitable due to its specific proprieties, namely high strength, chemical stability, and good thermal conductivity (Milnes et al., 2008). Crystalline rock formations prospected in northwestern Switzerland where the bedrock was assumed to be homogenous (Fischer, 2019: 296), where it was expected to be at a suitable depth of 600- to 1200-meters underground, and where mountain pressure was assumed to be high enough to compensate for disadvantageous fracture networks in the respective formation (Hadermann et al., 2014: 66).

Driven by the need to prove the feasibility of disposal, Nagra proposed an ambitious exploration program (Hadermann et al., 2014: 67). For the *in situ* exploration, Nagra submitted drilling applications in twelve sites in northern Switzerland (Fischer, 2019: 296). However, the planned drilling provoked opposition at these sites, causing administrative hurdles and delay: ultimately only seven out of 12 drills were conducted (Hadermann et al., 2014: 69–70). In addition to political challenges, the exploration drilling revealed the geological formation to be more complex than anticipated. It revealed that the crystalline basement in northern Switzerland was intersected by a large, 10- to 40-kilometer-long sedimentary trough—the so-called Permo-Carboniferous Trough (Fischer, 2019: 296). Seismic exploration undertaken between 1982 and 1984 confirmed the location of this trough (Hadermann et al., 2014: 71–73). Inaccurate assumptions about the subterranean increasingly disqualified crystalline formations in northern Switzerland for deep geological disposal. Consequently, the “Project Guarantee” synthesis report only partially fulfilled regulatory expectations when it was submitted in 1985: the evaluating governmental commission considered the high-level waste disposal project to be technically feasible but objected that “the proof of sufficiently extended rock bodies with the required properties” (Federal Council, 1988; cit. in Hadermann et al., 2014: 93, author's own translation) must still be provided.

The partial failure to prove feasibility made it impossible to expand the nuclear infrastructure. However, the acceptance of the technical feasibility of geological disposal allowed existing nuclear power plants to remain in operation (Fischer 2019)—while contestations continued with critical voices labeling the nuclear industry's promise a “myth” (Buser, 1988). To move forward, the exploration program shifted focus to Opalinus clay as the preferred option (initially among other sedimentary rocks) and as an alternative to crystalline granite formations (Hadermann et al., 2014: 146, 148). It appeared favorable mainly due to the clay formation's tectonically quiet bedding and its location in suitable depth in northern Switzerland—part of the geographical area that was already under investigation in the granite program. To foster the investigation of Opalinus clay rock properties, a rock laboratory was built at Mont Terri in the 1990s, a subalpine mountain range in the Swiss Jura where the clay formation is accessible relatively close to the surface (Bossart, 2017). Nagra launched a historically unprecedented 3D seismic campaign in northern Switzerland from 1996 to 1997 and an exploration drilling at Benken in 1998 (Hadermann et al., 2014: 148–149). Both exploration projects were publicly contested (Adler, 2023) and purportedly confirmed the favorable geological conditions, revealing “Opalinus clay with a thickness of 112 meters as a practically impermeable rock unit” (Hadermann et al., 2014: 149, author's own translation).

In December 2002, Nagra finalized “Project Opalinus” and submitted the according documentation as proof of the feasibility of nuclear waste disposal in clay rock formation (Hadermann et al., 2014: 150). The reframing of “Project Guarantee” to “Project Opalinus” had depoliticized nuclear waste disposal, shifting it from a problem of the nuclear industry to one that could be solved through geological disposal (see also Schürkmann, 2021). The documentation provided “proof” of the existence of “sufficiently extended rock bodies with the required properties” (Hadermann et al., 2014: 151, author's own translation). Nagra's proof of disposal was evaluated and finally, despite minor controversies (SFOE 2006), approved by the federal government on June 28, 2006 (SFOE, 2008: 19–20). The decision was key for the nuclear waste disposal program to advance as a matter of national responsibility for nuclear legacy waste. “Project Opalinus” thus strengthened confidence in deep geological disposal—evident in the codification of the concept as *the* solution for nuclear waste disposal under nuclear energy law (NEA, 2003)—and provided a basis for the selection of sites to be taken forward.

Despite the technolegal confidence, the project was complicated by local resistance to Nagra's parallel plans for site exploration for a small- and mid-level waste repository in Wellenberg, central Switzerland. There, local vetoes in 1995 and 2002 ultimately led to the rejection of the project (Krütli et al., 2010: 231–233). While low- and high-level waste programs had advanced separately with different planning horizons (Hadermann et al., 2014), the failure of the Wellenberg proposal had far-reaching consequences for Nagra's disposal program as a whole, marking the end of the local veto right and triggering a rather profound regulatory redesign (NEA, 2003; NEO, 2004). Furthermore, it provoked a reset of the nuclear waste management program by a Sectoral Plan in 2008, reconfiguring nuclear waste governance along with more participatory lines (Kuppler, 2017; Kuppler et al., 2023). As an established planning instrument for large-scale projects of national importance (Fischer, 2019: 303), the Sectoral Plan henceforth provided a broad framework for the institutionalization of local participation, favoring “relatively conflict-free” processes and negotiations between key stakeholders and potential host communities (Kuppler, 2017: 178). In turn, participatory site selection was favored by the emerging consensus for a nuclear phase-out following the Fukushima accident in 2011 and the corresponding national decision in 2017.

In any case, the Sectoral Plan confirmed Opalinus clay as the favorable host rock, highlighting the safety-related advantages of Opalinus clay, its “long-term confining capacity,” and its “quiet bedding” in northern Switzerland (SFOE, 2008: 19, author's own translation). The plan thus pushed forward the geological exploration of Opalinus clay formations in three stages. In the first stage (2008–2011), Nagra proposed geological siting regions based on safety and technical feasibility criteria. The proposal was reviewed and confirmed by the Swiss Federal Nuclear Safety Inspectorate (ENSI) and later approved by the Federal Council. It defined three sites (Jura Ost, Nördlich Lägern, and Zürich Nordost) for the potential disposal of high-level (as well as mid-/low-level) radioactive waste. In the second stage (2011–2018), Nagra advanced its exploration at these sites through a 2D seismic campaign to increase knowledge about the subterranean (Nagra, 2010). In 2014, ENSI confirmed the level of geological knowledge to be sufficient for a safety-related site comparison. Nagra carried out a provisional safety analysis and published its report to recommend two regions (Jura Ost and Zürich Nordost). The proposal was later rejected by ENSI and finally by the Federal Council, forcing Nagra to continue its exploration at all three sites—including Nördlich Lägern, which was previously excluded on technical grounds—in the third stage (SFOE, 2018; Nagra, 2014, 2016).

Site selection has continued in the third and final stage (2019–2024) with the subterranean exploration, intensifying through a drilling campaign at all three sites (Nagra, 2021a). Data from drilling were needed to complement data from the 2D and 3D seismic campaign—the latter of which had already run between 2015 and 2017—and to create a more comprehensive picture of the thickness, tightness, and composition of the host rock formation in northern Switzerland (Nagra, 2021b). To that end, Nagra submitted 23 drilling applications. Between 2019 and 2022, nine out of the 23 drills were conducted at the three sites, complementing data from the earlier campaign. In September 2022, the site comparison culminated in Nagra's proposal to select the site Nördlich Lägern, where the geological conditions appeared to be most favorable. In order to proceed with a deep geological repository at this site, Nagra prepared an application for a general license which was submitted to the authorities in November 2024. The federal government's approval of the application is expected around 2030 and can then be challenged only through an optional referendum at national level (NEA, 2003).

## Vertical projection

The recent drilling campaign set a new precedent for science communication. Nagra published an information brochure about the “deep drilling” in September 2018, clarifying the purpose of the drilling and the geoscientific investigation (Nagra, 2018). In December 2019, it released another brochure on “clay rocks and their contribution to radioactive waste disposal,” explaining why clay is considered a suitable host rock (Nagra, 2020a: 2; Nagra, 2019a: 2). Soon after the first drill rig moved downward into the Earth's crust, further science communication materialized in a series of events at the drill station in Bülach (Nördlich Lägern). The beginning of the campaign in spring 2019 was marked by an opening event organized exclusively for regional and national media representatives. Following this event, the drill site was regularly accessible to different stakeholders and local citizens in a series of open days and guided tours—first in Bülach and later at other drill sites as the campaign advanced. As I will illustrate, Nagra's approach to communication increasingly shifted away from providing information about geology, toward setting the stage for subterranean sensing and its embodied experience on the surface.

**Figure 1.**
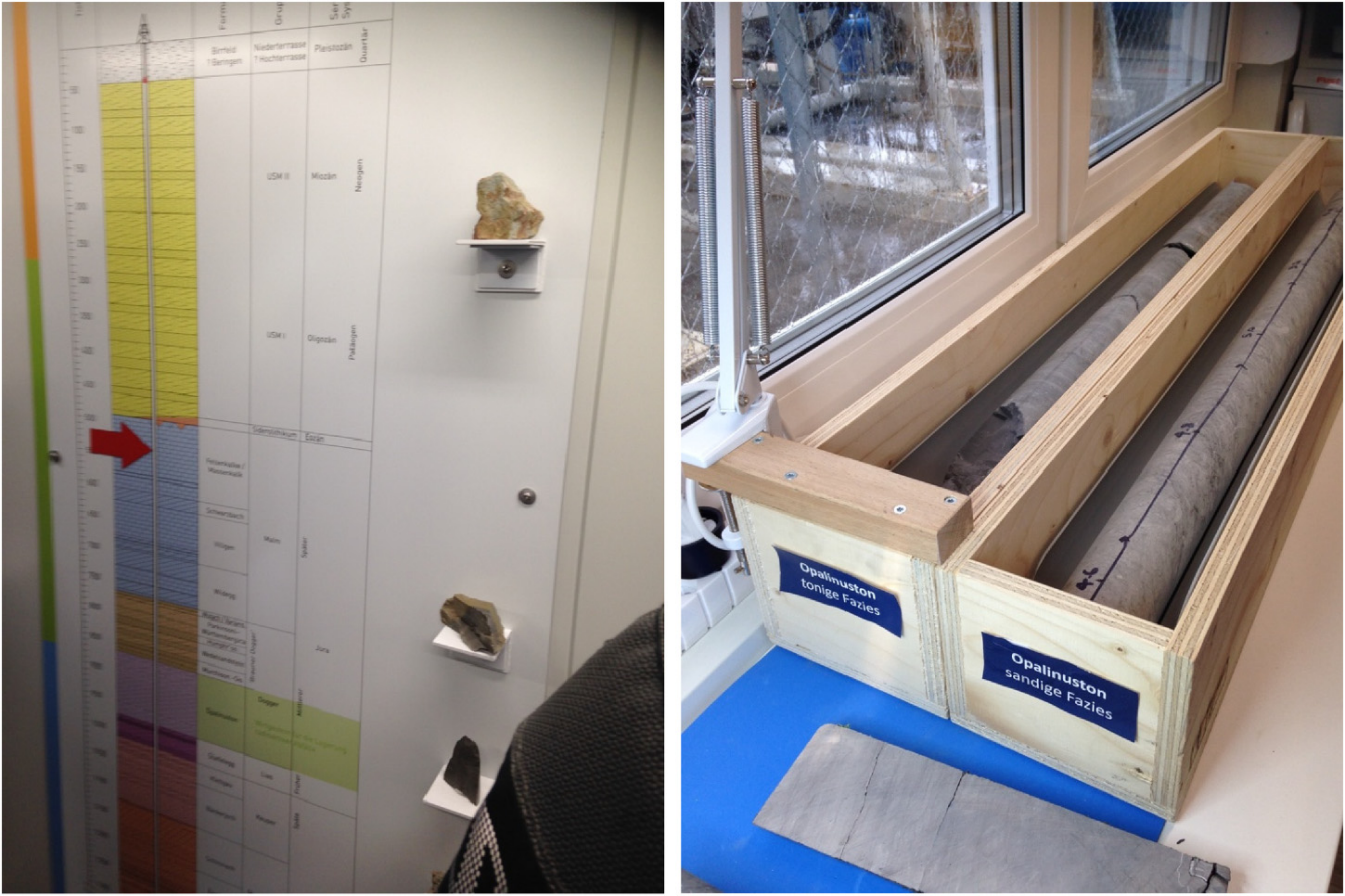
Vertical lithographic profile (left) and opalinus clay drill core samples (right) in the information pavilion in Bülach in spring 2019.

*Source:* Author's photo.

At each drill site, an information pavilion was built as an integral part of the drill station. At the top, a viewing platform open to public 24/7 enabled visitors “with a bit of luck … [to] experience how a drill core is pulled out of depth” (Nagra, 2019b, author's own translation). At the bottom, corrugated drill bits and a vertical drill profile decorated the entrance to a small exhibition, providing a glimpse of the technical means used for vertical drilling. Accessible on open days and upon registration, the exhibition offered visitors insights into the geological formation at the site. On one wall, a timeline illustrated how northern Switzerland had evolved over the past 300 million years as continents shifted and the paleogeography changed. On the opposite wall, the exhibition facilitated a view into the subterranean. In Bülach, for instance, a glass window allowed visitors to witness drill cores processed on the table after retrieval, as they were examined by geoscientists. Next to the window, a prognostic lithographic profile on the wall visualized the stratified constitution of the subterranean (Figure 1). Along with the stratification, exemplary rocks were displayed as objects representative of the respective strata underneath.

Opalinus clay drill core samples featured prominently inside the exhibition. These samples were arranged on a table in two 1-meter boxes alongside an explanation of its suitability for high-level waste disposal (Figure 1). The displayed samples served not only as visual representations but also as “epistemic devices” (Korff, 2005; Schürkmann, 2022) for visitors to know the Opalinus clay stratum as the drilling campaign's focus. They proved particularly effective in substantiating the stability of the respective subterranean clay stratum—manifest as a tangible rock property, often scrutinized by visitors inspecting such cores. Given their specific “texture” (Ballestero, 2019), drill core samples could be seen and touched, allowing an embodied experience of the subterranean. However, the drill core samples in the exhibition had little direct connection to the drill site as such: the samples originated from the Mont Terri rock laboratory and thus differed slightly in diameter from the cores retrieved at the site. Nevertheless, they came to represent what was to be explored: during visits, Nagra communication experts presented drill cores to visitors as simple rocks or as vaporized samples, making tangible not only subterranean geology but also the geoscientific process through which knowledge about the subterranean Opalinus clay would be produced.

Beyond enabling visitors to perceive the subterranean, the exhibition was instrumental in illustrating the presumed stability of the clay stratum at the site. The public demonstration of the stability of subterranean strata is a recurrent theme in various nuclear waste disposal programs, where critical publics tend to associate subterranean dynamics, instability, or fluidity with risk (Macfarlane and Ewing, 2006; Anshelm and Galis, 2011). In her study of site exploration in volcanic tuff in the United States, Allison Macfarlane (2003: 795–796) discloses how the responsible authorities have constructed a narrative of “dry is sound” and “dry is good” to satisfy critical environmental groups. The emphasis on the rock's dryness at the site does, however, not directly correspond with the safety-oriented evaluation of rock properties by geoscientists; to some degrees, geoscientists consider wet environments beneficial for reducing corrosion of the engineering barrier. Similarly, Richter (2017) demonstrates how “salt medium” is exhibited in the visiting center of the US Waste Isolation Pilot Plant (WIPP) to enact the subsurface host rock at the surface. As she has argued, guided tours at WIPP are “material aspect[s] of demonstrating … that the salt can contain the risks of radioactive waste.” In her case, the exhibition of salt and canisters—the natural and the engineered barriers—“are demonstrations of control, assuring visitors that there are many layers of protection from radiation” (Richter, 2017: 66–67).

In the Swiss case, the exhibition of clay in the information pavilion similarly served as a demonstration of control, with the nuclear hazard contained by Opalinus clay as the ultimate natural barrier in the disposal concept. The material properties of clay offered specific “political affordances” (Crippen, 2022) for convincing visitors of the stability of the subterranean stratum. These affordances of the clay lie in the relation between the communication expert and the sample itself; in how well the expert employs the tangible and affective properties of the core samples to produce desirable conclusions in the audience. In the exhibition, one affordance was realized through the strategic choice to demonstrate clay's ability to swell in contact with water. This behavior was illustrated on a screen in a moving sequence of an experiment of clay powder in contact *with* and *without* water, visualizing how clay expands in contact with water and thus caulks. The virtual experiment demonstrated the favorable rock property of Opalinus clay: its ability to seal nuclear waste in a suitable subterranean stratum. The material properties of clay therefore proved favorable for communicating science, making geological stability tangible in a dynamic way.

During a guided tour at the drill site in mid-May 2019, a Nagra communication expert similarly performed clay's favorable properties in a live experiment. He took a small, glass cylinder and put it on the table. He then took a gray powder, which he identified as “chemically purified clay” (author's own translation) called Bentonite, considered to be suitable backfill material in the multibarrier disposal concept.^2^ He poured it into the cylinder and then added water on the top of the clay. He then announced that he would reveal the experiment's results at the end of his presentation, which followed on the screen. Time passed quickly as the audience listened to his discussion of the deep history of Opalinus clay, its favorable rock properties, and the construction of the drilling site. Upon finishing his presentation, the expert finally turned his attention back to the cylinder. He lifted it up, letting the clay powder fall out. However, a tiny layer of clay had resorbed the water and remained stable in the middle of the cylinder, effectively sealing the water in above it and preventing it from seeping out—*quod erat demonstrandum* (that which was to be demonstrated). The experiment allowed him to demonstrate that clay expands in contact with water and thus assumes a stable form. In the expert's performance, as we learn, clay emerged as what Barry (2013: 142) has described as an “informed material” that is bound up with information about its favorable material properties. The experiment was then not simply a matter of accurately describing the material's properties but of assessing its ability to fulfill the regulatory requirement as a host rock for the long-term containment of nuclear waste.

The tour guide appeared proud of his experiment's success, saying, “the experiment works brilliantly for visiting school classes.”^3^ However, his performance was not entirely straightforward, as he clarified in our personal conversation after the tour. He had to train himself to figure out how the experiment works best and how it could be optimized. He explained that it was critical to throw the water into the cylinder rapidly enough to ensure enough clay powder is awhirl and saturated with water, allowing it to sustain a stable layer at the end. Also, the diameter of the cylinder matters and reducing it would allow him to increase the success rate of his performance. As he revealed, however, the experiment was fragile and therefore not always suitable; whether or not he decides to enact clay as a stable stratum in the glass cylinder also depends on his expectation of how his audience will react. For example, he had excluded the experiment from his program under other circumstances, as its potential failure bears the risk of provoking a controversy. While the experiment allowed the expert to demonstrate the stability of clay, its performance was politically conditional.

## Horizontal depth

The availability of drill cores from all three disposal regions created a strategic moment for science communication. Given the Covid-19 pandemic at that time, Nagra's communication unit organized an online event for media representatives in early November 2020 to share initial findings. Nagra opened the core storage hall where drill cores were arranged in sequence for geoscience investigation and analysis. The Deputy Head of Public Relations opened the event, stating that “rock samples have been arranged for regulatory oversight. … It is thus also a good opportunity for the media to look over our shoulders” (Nagra, 2020b, author's own translation). The moderator of the event then directed the attention to the Head of Geology and Safety, announcing his input on “pure geology.” Holding a drill core sample, the geologist addressed media representatives and explained that the retrieved drill cores are “mapped, documented, recorded, scrutinized, and interpreted” in order to “assess the entire geology of this place.” To substantiate his explanation, the geologist engaged the drill core in his hand not simply as an epistemic object but as a scientific one produced in the course of the geoscience investigation.

Before digging into the arranged drill core sequence, the geologist moved into the corner of the storage hall, where drill profiles from three boreholes were displayed on the wall. He explained that drill cores were measured and scanned, with the three profiles summarizing “the scientific result of the core recording and the diverse geophysical investigations.” These “preliminary lithography plots” provided evidence for the ongoing data analysis, capturing the material properties of the geological formation at specific sites and at different depths. As the geologist highlighted, “the main objective of our investigation” was to illustrate the significance of the Opalinus clay stratum in the Swiss disposal design—the “gray section” visible in the plot on the wall. He explained: “This deep geological repository is packed or protected by a thick layer of [Opalinus] clay, which ensures that waste remains contained.” He continued to elaborate that Nagra's seismic investigation had already revealed the subterranean “geometry,” that there is “enough space” for the construction of a repository, and that the drilling campaign revealed “an extensive, thick and dense Opalinus clay layer in all three sitting areas.” To substantiate his claim, he had prepared three core samples from various drilling sites for comparison, stating, “you can see that the Opalinus clay really does look very, very uniform.” The geologist had moved forward to a table with the three sample cores whose gray appearance served as a visual metaphor for the homogenous stratum. His findings were positive news in the context of a contested nuclear waste disposal history and gave direction to the continuation of the disposal program. In fact, Nagra's application for a general license would ultimately require a more detailed and nuanced presentation of the scientific investigation and results for the regulatory authority's review by the end of 2024. For the media event, however, it was enough to tell a simple story that made the stability of the Opalinus clay tangible and transparent to the public.

**Figure 2.**
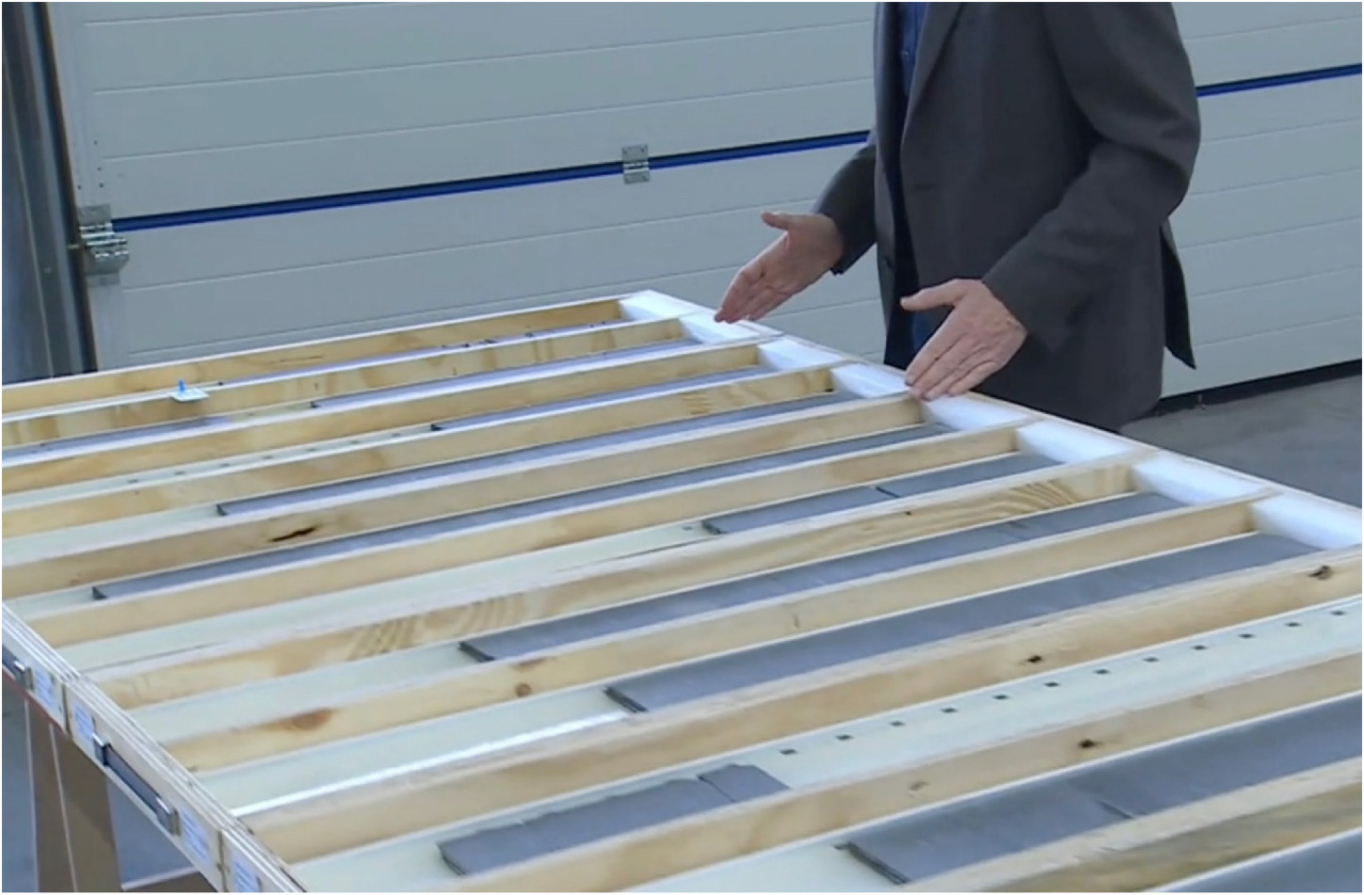
Nagra head of geology and safety guiding media representatives through the Bülach drill core sequence during an online even in November 2021.

*Source:* Author's screenshot.

The geologist moved on to guide his audience through the sequence of drill cores arranged in horizontal rows in the storage hall. Reference for his presentation was again the Bülach drill core—shiny slices of cores in one-meter boxes juxtaposed in a well-ordered arrangement (Figure 2). The organization is telling. As Wrigley (2023: 10) explains in her discussion of a drill on the Kola Peninsula, cores “have become material containers of information … laid out in neat little rows that fit the shattered pieces together to produce a visual representation of the underground at the surface.” The horizontal arrangement of “perfectly vertical and straight” cores, as she argues, is instrumental to upholding scientific objectivity and the presentation of the scientific exercise as “being controlled and untainted” (Wrigley, 2023: 8). In turn, cores neatly arranged “suppress the messiness and discontinuity of the hole itself,” obscuring that drill cores do not emerge as vertical lines (Wrigley, 2023: 10). In fact, cores from Bülach were also not drilled along with an exact vertical line as its neat geometric arrangement in the hall suggested, but through what experts call a “natural curvature” (Wrigley, 2023: 10). Highlighting the technical challenge of drilling along a vertical line is not an attempt to undermine the accuracy of the geoscience. Rather it is an attempt to demonstrate that the geometric arrangement of drill cores at the surface conditions our perception of the subterranean and becomes constitutive of ideas about scientific objectivity in both science and public discourse.

For the geologist, the neatly arranged drill core display also set a horizontal trajectory for situating the core in a deep time past. What followed was, as he put it, a “leap in [deep] time” (Nagra, 2020b, author's own translation), leading the geologist and his virtual audience to the start of the drill core sequence: a 200-million-year-old rock retrieved from about 1000 m depth. By walking along the drill core, he situated different segments in specific imaginaries of the paleontological climate and the geophysical processes that formed sediments at the time. The geologist reached the Opalinus clay formation after about 60 meters, a distance equivalent to 60 million years “as one meter of sediments was deposited in about a million years.” He then continued to explain the “really drastic change” evident in the drill core sequence: “What comes from here, the next 100 meters, have been actually deposited in a million years. That means we have about a hundredfold accelerated deposition.” As he elaborated, the accelerated deposition rate was the result of tectonic processes and an increase in the sea level, leading to clay sedimentation at greater water depths. The consequence of these shifts was “a regional stratum” of Opalinus clay that is, as he emphasized, unique to northern Switzerland, parts of southern Germany, and France. Moving forward, he passed through the Opalinus clay sequence, purportedly formed at an extremely high deposition rate, building a 110-meter-thick stratum with favorable rock properties. He reached the end of the stratum marked as the “Opalinus top” summarizing, “we saw a 100 meters of Opalinus clay deposited in a million years. So, with a very very high rate of deposition and now we go back to *normal*, if you will. Now we have again about one meter of sediment per million years from here” (emphasis added).

**Figure 3.**
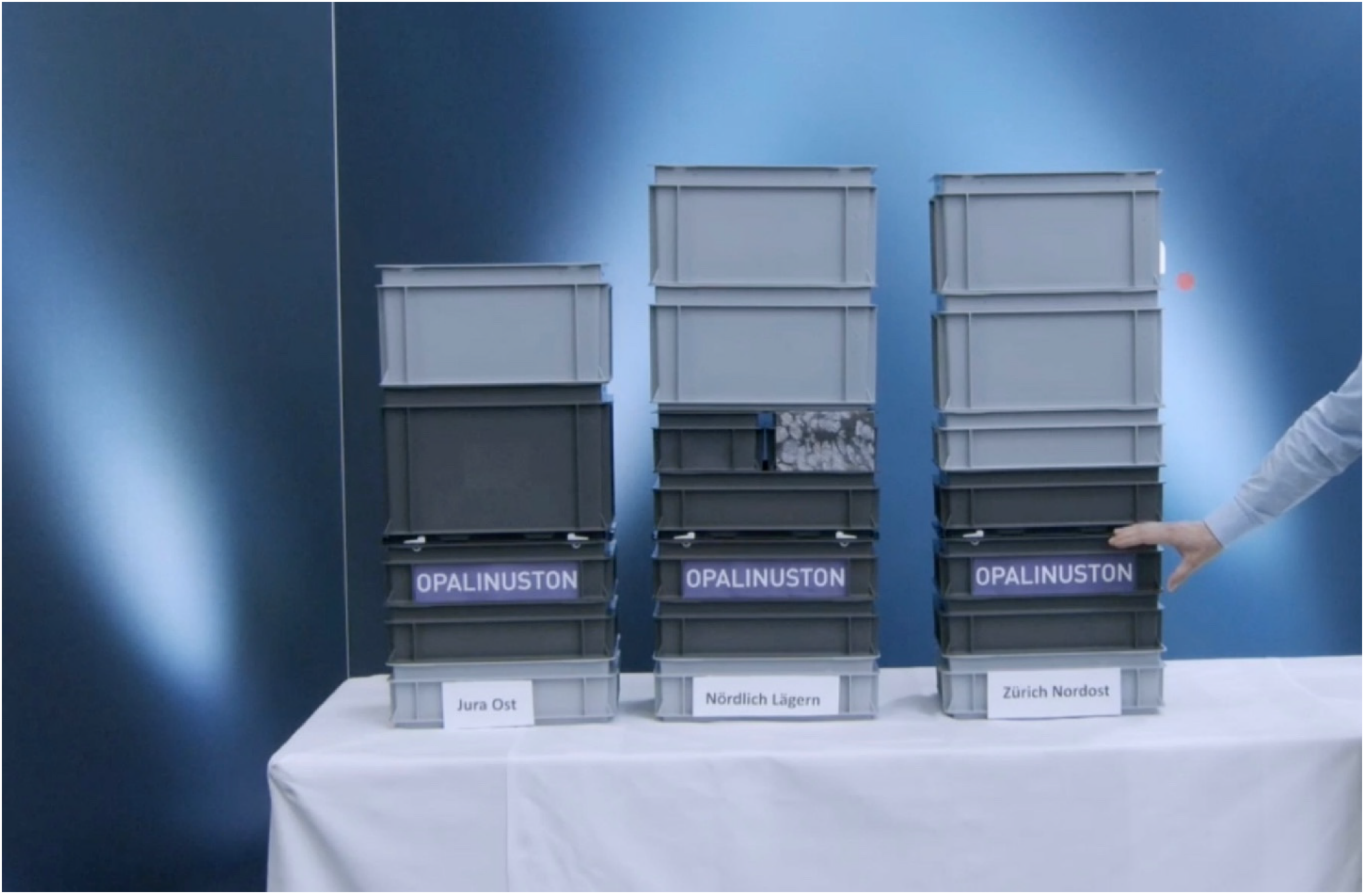
Nagra deputy head of public relations summarizing the geoscience findings for media representatives during an online event in November 2021.

*Source:* Author's screenshot.

The geologist enacted the Opalinus clay stratum as a geological exception produced by Earth-building processes in a paleontological period during which time arguably slowed down significantly. This Opalinus clay stratum was reified through the later editing of the event recording. In the version that appeared on Nagra's Youtube channel, the initially lengthy paleontological description of rocks predating the clay formation was cut in favor of a short sequence starting at the “Opalinus bottom”—followed by the reference to the abrupt change in climate conditions, the emergence of an ocean in which clay then sedimented, and the description of Opalinus clay's favorable properties as a host rock—and culminating at the “Opalinus top” (Nagra, 2020b). The Youtube version henceforth focused viewer attention on the Opalinus clay stratum as it was of political interest for the deep geological disposal project. It testified to what media scholars have highlighted since the “geological turn” in their field: that media mediate “our relation with the Earth” (Parikka, 2015: 13). It also reveals the interplay of visual and material practices through which clay is demarcated as a stratum and staged as the ultimate barrier for deep geological disposal (Figure 3).

In both versions of the event, the geologist's presentation of the “pure geology” was complemented by the Deputy Head of Public Relations who provided a simplified summary of the “current state of knowledge” at the end (Nagra, 2020b, author's own translation). The latter explained, “as a trained geologist myself, it is always fascinating for me to see how my colleagues keep a close eye on the underground in the siting regions.” He emphasized that summarizing is paramount and suggested doing so by means of what he called a “haptic approach.” To that end, he made use of standardized plastic boxes as an “allegory for geological strata,” with dark gray boxes representative of geological strata capable of containing radioactive waste in the long term, and light gray ones for those less capable. To start his demonstration, he placed a miniature yellow nuclear drum into the Opalinus clay box, closed it, and stapled it vertically. Building three parallel stacks from the bottom to the top, one for each site, he presented different stratigraphic profiles as simplified models of how the subterranean is constituted in different regions. These boxes thus allowed him to build the subterranean in the three regions as a stratified sequence of boxes. This visual helped him to illustrate the stability of subterranean strata upon which he concluded: “In summary, one can say that in all three regions we have an area of Opalinus clay that meets the requirements and allows safe construction and operation of the deep geological repository and precisely this long-term required containment.” His haptic approach had not only allowed him to simplify the rather complex geoscience investigations but to demonstrate Opalinus clay's stability and ability to contain nuclear waste, regardless of the site.

## Voluminous clay

The end of the drilling campaign in spring 2022 once again inspired Nagra's communication team to open up to the public. As this was a time for celebration, Nagra commissioned event specialists to design an exhibition in collaboration with its own communication experts. The resulting exhibition was aimed at the general public, to whom it was open for two days, and at various local, national, and international stakeholders, who were invited and guided by Nagra. In view of the upcoming site selection, the aim was no longer simply to display the Opalinus clay cores obtained during the drilling campaign but also to create a space for dialogue—and “to deal with questions and criticism” (Wolgensinger, 2023, author's own translation). To this end, the exhibition now pushed forward Nagra's science communication toward what Zimmer (2022: 37) has described as “emotion management,” moving away from the dissemination of information toward the communication of simple messages through aesthetic means. As one of the designers later put it during our discussion in 2024, the exhibition was designed around the key finding—the favorable properties of clay as a host rock and, essentially, its inertness.

**Figure 4.**
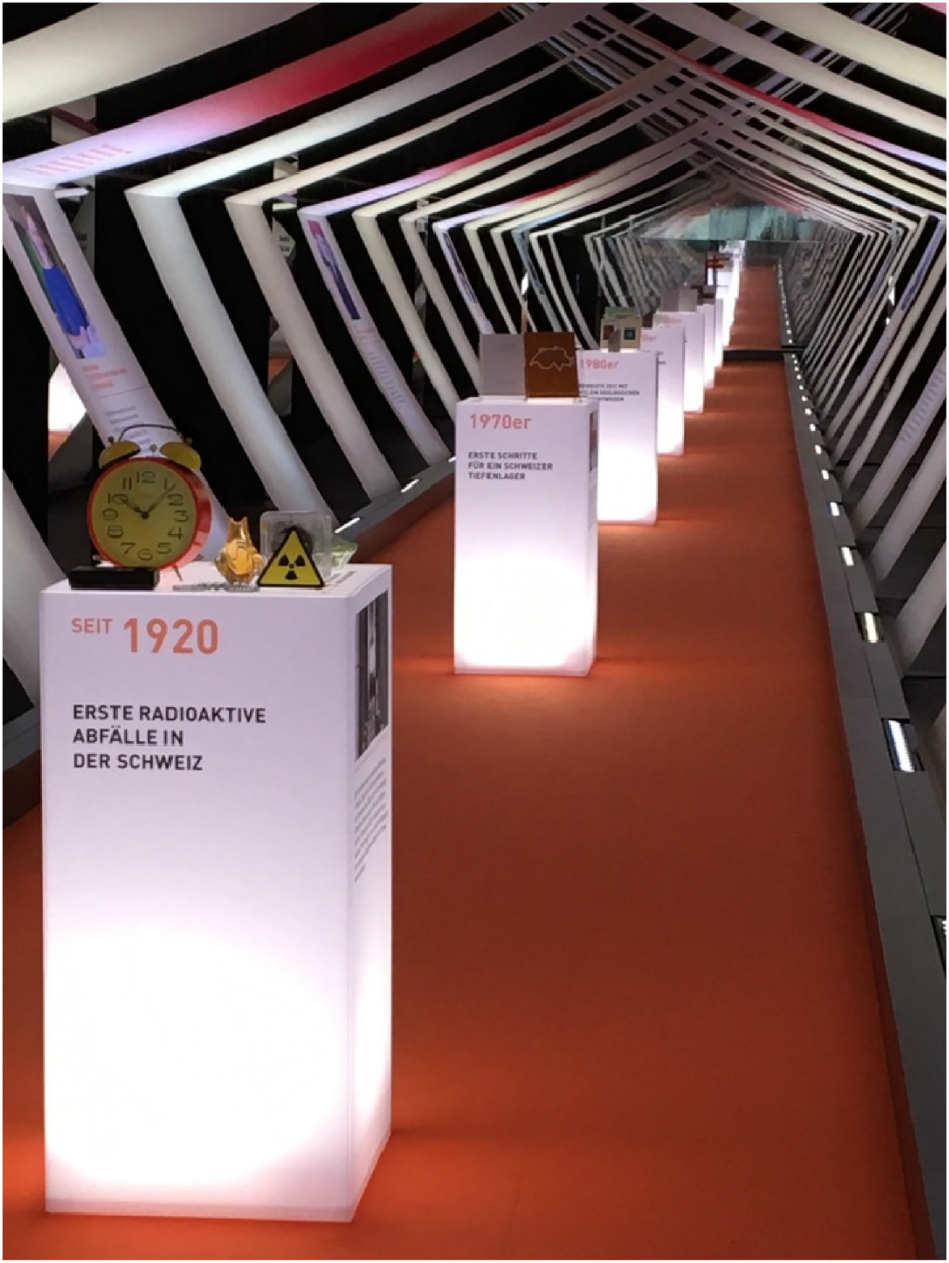
The opening tunnel to the exhibition “Nagra—From deep into the future,” Windisch, Spring 2022. A first pedestal depoliticized nuclear waste, displaying it in form of early everyday objects, rather than as nuclear energy products.

*Source:* Author's photo.

The exhibition marked the culmination of Nagra's public performance of subterranean strata. Titled “Nagra—From the Deep into the Future,” the exhibition invited its audience on a journey through human and geological history: visitors entered a long corridor, designed as a tunnel, along which Nagra's 50-year history was illustrated by a sequence of pedestals, each dedicated to key historical moments. These pedestals made a rather complex and contested history accessible through short descriptions and tangible through a set of bureaucratic and geological artifacts relevant to the respective historical moment. It also included one pedestal dedicated to the initial project advanced in crystalline granite in the 1980s and another to the subsequent Opalinus clay project in the 1990s—each materially represented by a granite and clay drill core on the top of the respective pedestal. The narrative along with this historical trajectory was open to the dynamic process, taking into account the failure of the disposal plans at Wellenberg and the subsequent participatory turn (Kuppler, 2017; 2017). Nevertheless, it remained partially apolitical, notably ignoring the severe antinuclear protests that shaped nuclear waste policies and programs, especially in the 1970s and 1980s (Buser, 1988; Häni, 2018). Instead, the exhibition built a technical storyline, suggesting that an initial period of “great geological findings” (author's own translation) in the 1980s later resulted in the finding of “Opalinus clay as the key to the disposal proof” (author's own translation), and culminating in the federal government's approval of Nagra's proof of disposal in the early 2000s (Figure 4).

**Figure 5.**
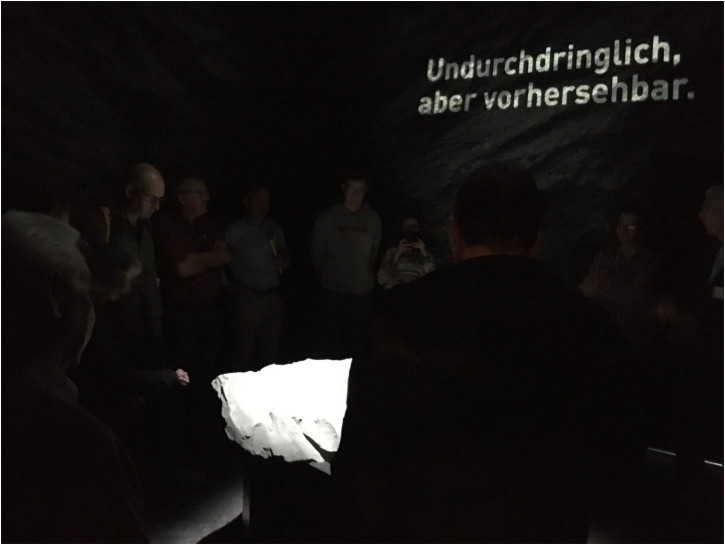
Opalinus clay boulder in the exhibition “Nagra—From deep into the future,” Windisch, Spring 2022.

*Source:* Author's photo.

At the end of the tunnel, three red fingerposts on the final plinth indicated the direction to each of the three potential disposal sites, symbolizing the openness of the siting process. An opening at the end of the tunnel led visitors into a dark, tomb-like room—labeled the “room of silence” (Nagra, 2022: 11) by its designers—with a boulder staged prominently under a spotlight (Figure 5). The boulder consisted of a massive piece of Opalinus clay rock brought from the Mont Terri rock laboratory. Placed in the center of the room, it allowed visitors to relate to the rock through observation as well as touch. Spheric sound engulfed the site, generated at 432 Hz—the frequency of cosmic harmony that is believed to promote self-healing of the human body (see Rosenberg, 2021). Aphorisms and platitudes projected on the surrounding dark walls waxed and waned, declaring clay's favorable properties as host rock: “175 million years of (cultivated) boredom; Time stands still; Stone-old, stone-gray; Closed—and still a jewel; No room for interstices; Inner values that do not leak out; Impenetrable, but predictable; A rock speaks—and science listens” (author's own translation). The exhibition was designed to facilitate a sensual encounter with Opalinus clay and seemed to create what Van Geert (2019: 137) refers to as “the wow effect.” Nevertheless, the meaning of the boulder was not set in stone: during certain visits, the tomb and boulder may have motivated tour guides to rely on a more technoscientific introduction of clay's central rock properties. The boulder could thus be enacted in both aesthetic and epistemic terms, in line with clay's ambivalent political affordances.

**Figure 6.**
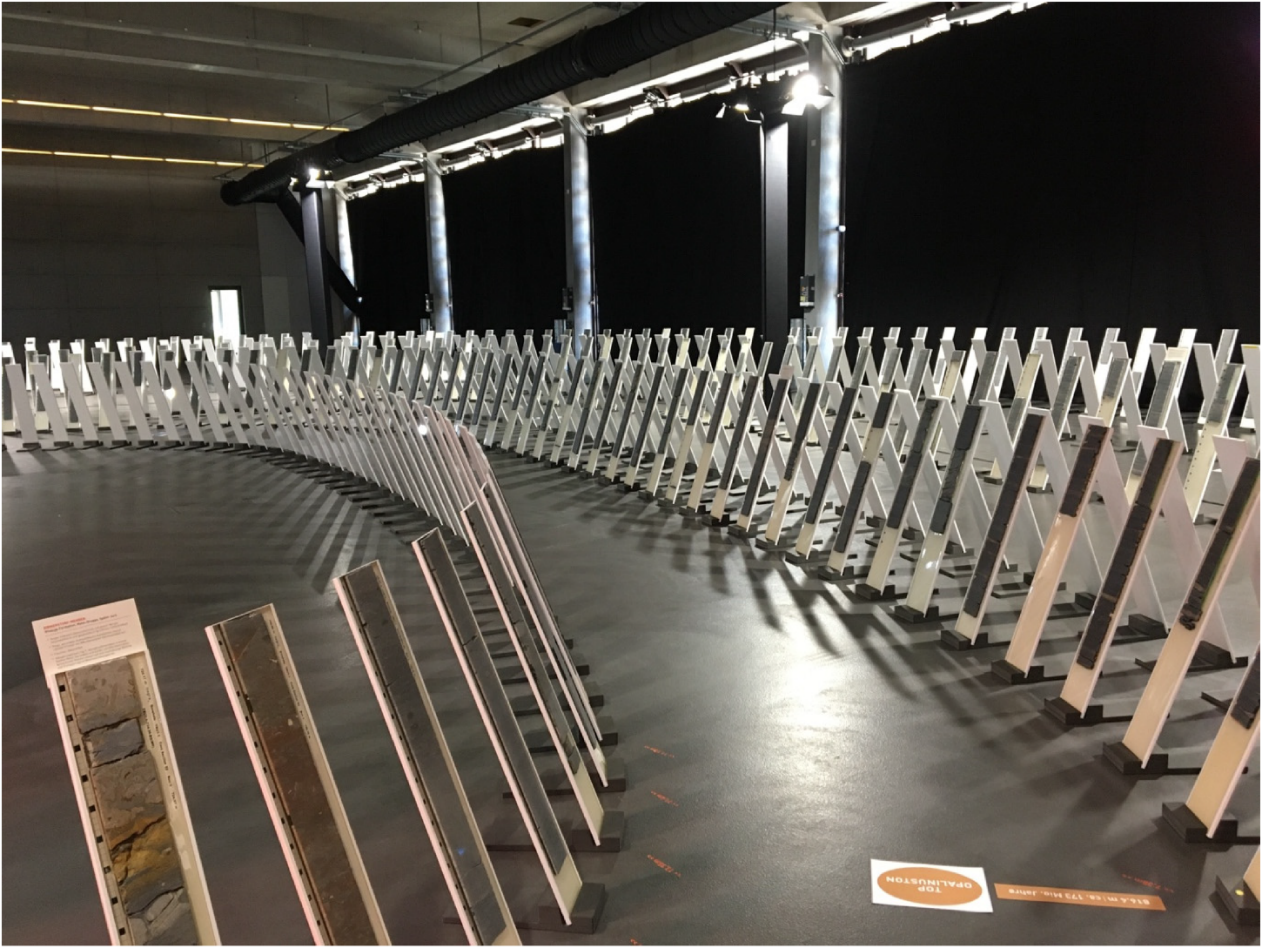
Opalinus clay drill core in the exhibition “Nagra—From deep into the future,” Windisch, Spring 2022.

*Source:* Author's photo.

Visitors left the room through a black curtain and entered the drill core exhibition (Figure 6). Three gates or arches framed the space, built to the scale of future repository shafts. A sign identified the drills, one for each siting region with information about the respective drilling period, drilling depth, and the size of the Opalinus clay stratum—with the Bülach drill in the middle. Behind the gates, drill cores were erected as nearly vertical slices one after another. Thin sections were cut from the middle of drill cores and then impregnated with resin—as what experts call “slabs.” The resin impregnation stabilizes the thin section, protecting the clay from oxidation and lending it a translucent appearance. In contrast to their neat arrangement in the storage hall (Figure 2), drill cores in the exhibition were no longer organized along with straight lines but rather displayed as meandering paths. One might be tempted to argue that the spatial arrangement of the drill cores potentially undermines the claim to scientific objectivity—a perspective that the geometric outline of 1-meter boxes in the storage hall sustained. As one of the designers remembered during our discussion in 2024, some geologists were indeed puzzled by the unusual arrangement of the cores. However, the purpose of the exhibition was obviously different from the storage hall display. The meandering staging of the vertical drill cores was of aesthetic appeal, facilitating an embodied experience of a voluminous Opalinus clay stratum as one walked along with the path.

In contrast to the drill core in the storage hall, this exhibition did no longer included the full drill sequence but focused primarily on the Opalinus clay stratum, framed by a few core samples from the strata above and below. The Opalinus clay stratum was bounded by two lines on the floor, one at the start marked as “Opalinus clay bottom” and one at the end marked as “Opalinus clay top.” As one of the Nagra communication experts later put it: “When faced with the task of showing 200 million years of the Earth's history, we were spoilt for choice: 6000 meters of drill cores lay before us! Which ones would we choose to illustrate our journey to the siting proposal? How to combine scientific meticulousness and vivid aesthetics? This was a balancing act” (Nagra, 2022: 12). The balancing act she refers to was two-fold: on the one hand, it was about making a pragmatic selection of the scientific objects produced during the drilling campaign. On the other hand, it was about a strategic decision whether to produce an exhibition of primarily scientific or aesthetic appeal in preparation for the upcoming site selection. The choice for the aesthetic arrangement of scientific objects in the exhibition finally enabled visitors a more sensorial and emotional encounter with a voluminous Opalinus clay stratum.

In the exhibition, therefore, the drill cores were presented not simply as evidence of a rather complex geoscientific argument, but as evidence of the existence of an extensive host rock formation required for deep geological disposal. The Nagra CEO was quoted in early March 2022 at a media event organized in conjunction with the exhibition, saying: “Never before had the ground anywhere in Switzerland been so well examined … The picture of the subsurface was now complete” (Berger, 2022, author's own translation). There were apparently no surprises for Nagra as the boreholes confirmed the assumptions about the site suitability, evidenced by the visual appearance of Opalinus clay. The Nagra CEO articulated the homogenous gray facies of the “unspectacular” and “boring” clay as a metaphor to emphasize the stability of the Opalinus clay stratum. However, the metaphoric homogenization overshadowed the fact that facies differences existed and provoked visitors’ and geoscientists’ curiosity (see e.g., Lauper et al., 2021; Zimmerli et al., 2024). Nevertheless, the gray facies that characterizes the Opalinus clay core samples from all three sites highlighted the favorable properties of clay rock, regardless of the site.

However, the aesthetic bias did not exclude communication about the ongoing geoscientific investigation. As the Nagra CEO declared at the media event, the drilling had seemingly completed the picture of the subterranean: “400 drill cores … have provided researchers with information about the geology over the past decades” (Wolgensinger, 2023, author's own translation). “Now the big arithmetic begins. We have a lot of data. We have the experts’ hypotheses. Now we’re pulling it all together and in fall we will be ready for a joint decision” (SRF, 2022, author's own translation). The Nagra CEO hinted at the complex geoscientific analysis undertaken by his institution and the geoscience research community more widely. As I later learned in conversation with one of Nagra's communication experts, a fourth room dedicated to the geoscientific analysis had been considered in the initial designing phase of the exhibition but was ultimately abandoned. Instead, a monitor placed close to the exit of the exhibition provided animated insights into the in-progress analysis of the geoscience data. A short sequence from the data visualization lab, the so-called V-Lab at Nagra's headquarter, was played at the periphery of the exhibition. It revealed that drill core data and visual scans have been integrated in a dynamic 3D-model in which data gathered through seismic and drilling could be selectively retrieved by geoscientists at work and visualized for disciplinary and interdisciplinary analysis. It highlights the way in which the more aesthetic claim of a stable clay stratum is authorized in relation to the parallel production of scientific knowledge—ultimately to promote public confidence in geology.

## Nuclear strata

In a press conference held in the Swiss capital on Monday, 12 September 2022, the Nagra CEO announced Nördlich Lägern as his organization's preferred site for nuclear waste disposal. While open to media representatives only, the conference was set up as an online livestream for all those not accredited by the federal media center where the conference took place. Once the microphone was handed over to the Nagra CEO, he explained, “today, we have reached an important milestone in the deep geological disposal project, as we have the opportunity to announce the site for the disposal of all types of nuclear waste—for a so-called combi-repository. It is encouraging that it is an unequivocal decision: *Geology has spoken!*” (SFOE 2022, author's own translation, emphasis added). In fact, his emphasis on geology remained ambiguous, as it could refer either to the geosciences that had created a knowledge base for decision-making or to the clay rock itself.

**Figure 7.**
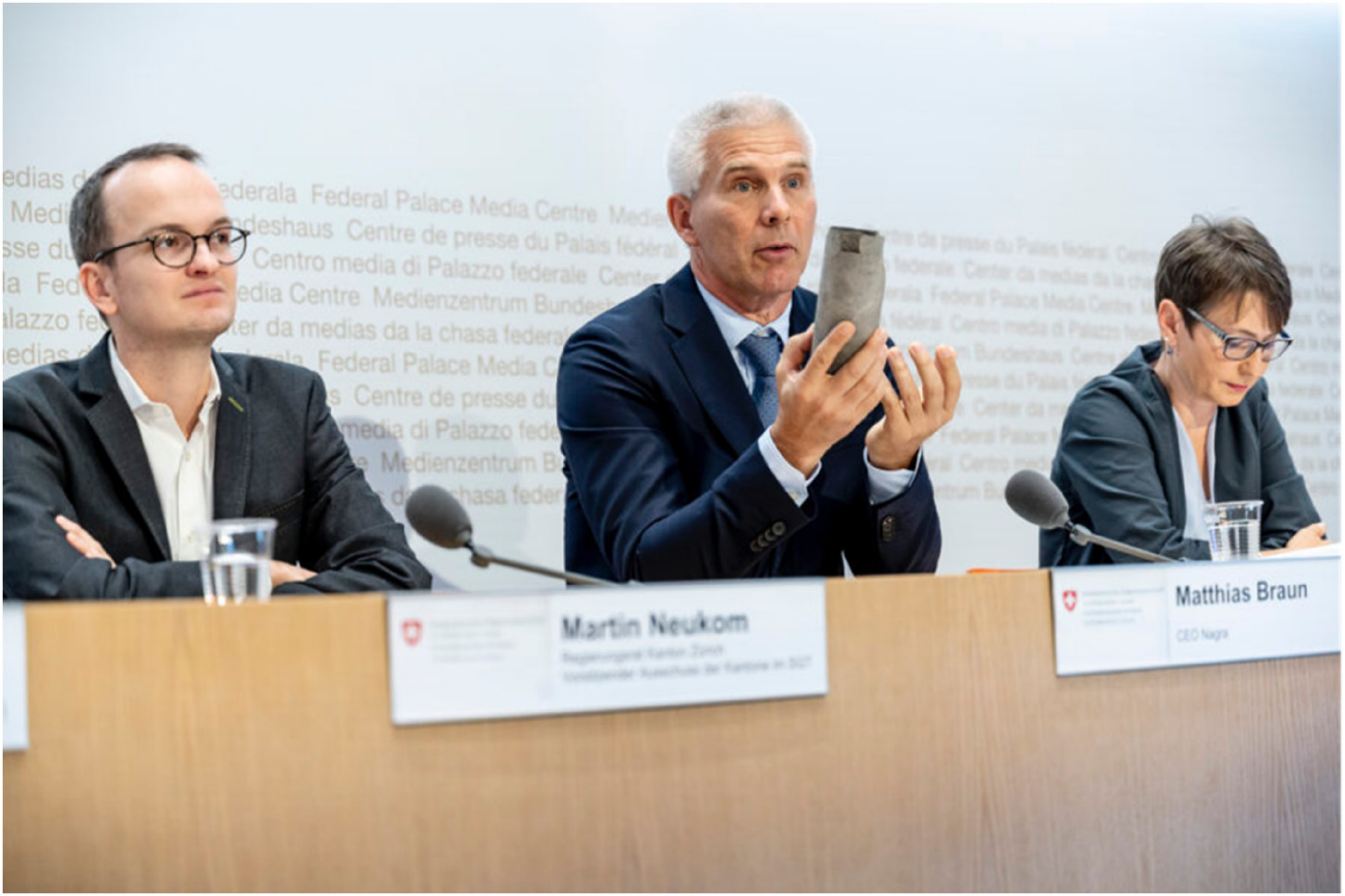
Nagra CEO at the press conference on 12 September 2022.

*Source:* Author's screenshot.

He then gratefully acknowledged all those who had contributed to this achievement—his geoscience team, federal and local government authorities, the local communities, as well as those who allocated land for the seismic and drilling campaigns. He then shifted his attention to a drill core from the Mont Terri rock laboratory (Figure 7), which he presented to his spectators as he continued to explain: “The heart of the deep repository is this gray, unremarkable rock: the Opalinus clay. It is about 175 million years old, unremarkable, gray—boring from a geological perspective. Time almost stands still here. Knowledge about its history over the last 175 million years makes us confident in making good prognoses for the deep future.” He continued to explain the three essential properties qualifying clay as a host rock: its density, its ability to absorb radionuclides like a “magnet,” and its “self-healing” capacity in the unlikely event that it cracks. He went on to clarify that the Opalinus clay stratum is the most important geological barrier in a “modern safety concept that builds on redundances”—or the “onionskin model,” as he put it. As he claimed, such a barrier is luckily available in sufficient thickness at all sites—making sure that the modeled radiation dose will remain much below the prescribed threshold in all three regions in the future.

**Figure 8.**
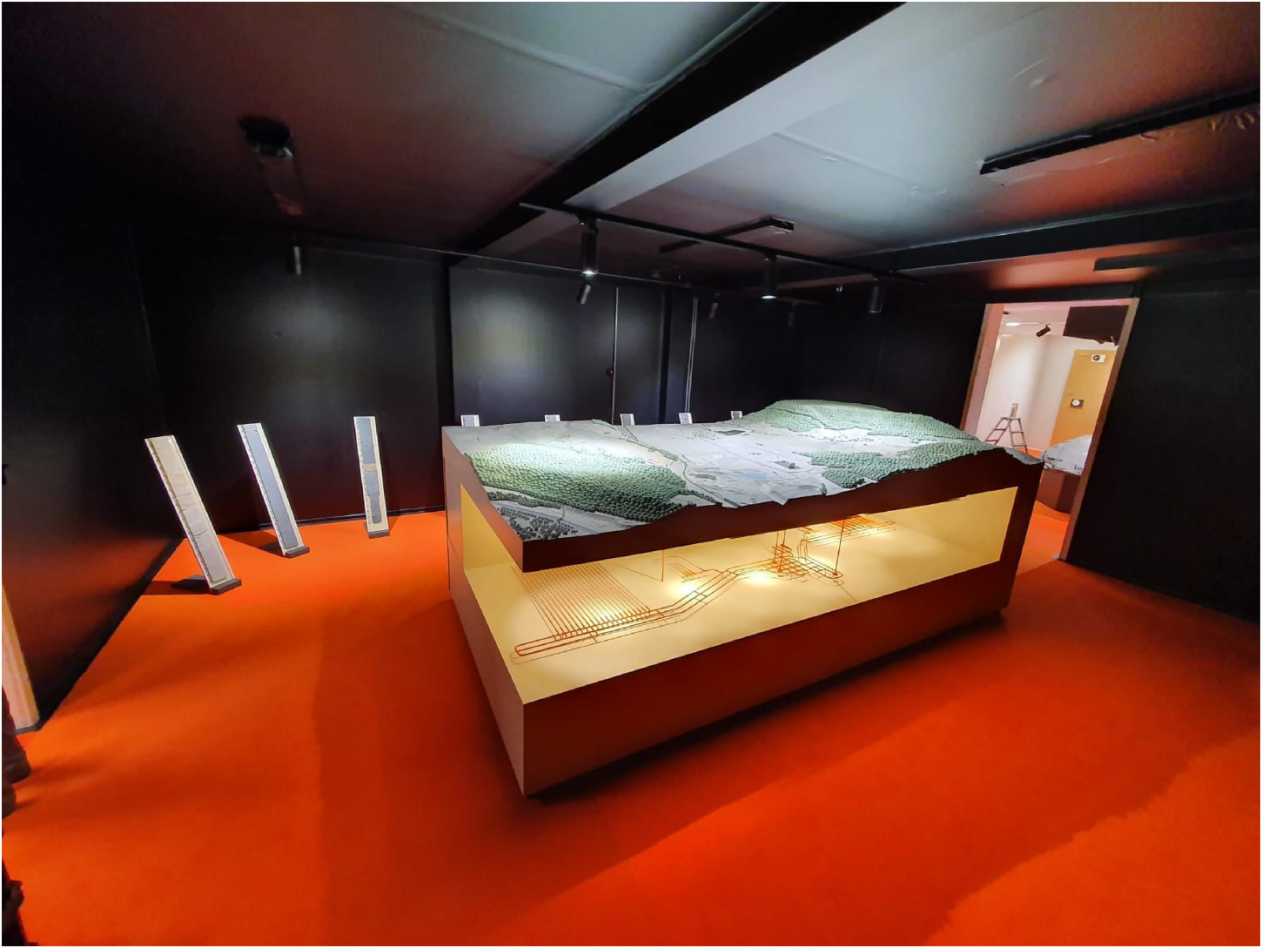
3D model of the deep geological repository in the information pavilion in stadel, September 2022.

*Source:* Nagra.

However, the main purpose of the event was not to justify the legally established deep geological disposal concept but to substantiate Nagra's site selection. The CEO highlighted differences between the three considered sites. For illustration, a stratified cube appeared as a “stack of stones” on the slides behind him as a “simplified” visual representation for the subterranean at all three sites, with the Opalinus stratum of “about 100-meter thickness” highlighted in gray, surrounded by lighter colored strata. As he elaborated, “in the Opalinus clay, you can find traces of old water, very old water. This water is the oldest in Nördlich Lägern. And here nature has already performed an experiment *for us*. It has tested containment, even over relevant periods of millions of years” (emphasis added). The CEO's discursive framing of Opalinus clay underlines a certain ambivalence of how the emerging geosocial relationship is conceived: deep geological disposal through passive safety builds on a clear-cut divide between the social and the geological, while simultaneously integrating Opalinus clay into the sociopolitical realm of the disposal project. It reveals that deep geological disposal is not only about, as Schröder (2016: 698–699) demonstrates, the “delegation” of responsibility to the host rock, but more fundamentally about the necessary “distribution of competences” between humans and nonhumans for handling nuclear waste.

Underlining clay's competencies, the Nagra CEO elaborated on the key advantages of the Opalinus clay stratum at the site Nördlich Lägern, referring to the slides behind him. He explained that the Opalinus clay stratum at the site Nördlich Lägern is best in terms of “quality,” as the distance to the surrounding water-bearing layers is comparatively bigger; it is most “flexible,” as it contains the largest “quiet and undisturbed” area available for the placement of a deep geological repository; and, it offers the most “stable” conditions thanks to the largest distance to the surface potentially affected by fluvial and glacial erosion. With reference to the quality, flexibility, and stability of the Opalinus clay stratum in Nördlich Lägern, the Nagra CEO thus simplified the main criteria for site selection. The vocabulary was deliberately chosen for the press conference to nail down a rather complex site comparison in three simple and positively connotated terms. “Stability” thereby acquired a new, more specific meaning when articulated as only one of three key properties of the Nördlich Lägern clay stratum. However, from an analytical perspective, the stability of a bounded Opalinus clay stratum appears to be a consequence of its categorical demarcation from the existing dynamic aquifers above and below, as well as from the geomorphological dynamics eroding the surface in the long term. Opalinus clay was thus no longer simply enacted as an existing stable subterranean stratum but as one that would remain stable over time. In other words, stability appeared as a spatial as well as a temporal property of the Opalinus clay stratum in Nördlich Lägern, qualifying it as the favorable host rock for deep geological disposal of nuclear waste in the future.

In the wake of the national press conference, the Nagra CEO repeated his message at series of events followed in the Stadel municipality, now in the spotlight at the site Nördlich Lägern. On September 17, the Saturday after the press conference, the Nagra communication team opened a new pavilion close to the municipal center, at the site of a drill that had been ongoing from January to July 2021. In contrast to the previous pavilions at the drill sites, the Nagra communication team had decided to forgo information boards and videos, with the exception of a brochure rationalizing the site selection. In one room, the new pavilion integrated the Opalinus clay boulder from the previous exhibition. It seemed a rather mundane testimony to the now well-known host rock. Yet the apparently “inconspicuous gray rock” had become Nagra's “unsung hero,” celebrated for its competence in containing nuclear waste (Nagra, 2022: 11). In another room, 1-meter thin sections of cores from the Bülach borehole were again displayed along the wall, illustrating the internal structure of the Opalinus clay formation—alongside other core sections from above and below. However, the focus in this room no longer seemed to be on the host rock, as evidenced by the fact that only three out of ten cores were actually Opalinus clay. As a Nagra expert explained during our informal chat on the spot, the line of core sections had been selected by the geologist, whom he had asked for a selection of “beautiful cores.”^4^

As the disposal project had advanced, the centerpiece of the new pavilion was now a new attraction—a three-dimensional model of the siting region. The model depicted the surface topography of the region and the proposed design of a deep geological repository (Figure 8). For the Nagra communication expert present at the exhibition, it described a preliminary structure and design of the planned repository with one section for high-level waste and another for mid-/low-level waste, with a few shafts connecting the facility to the surface. In parallel, the model developed as an anchor for visitors to connect their lived experience on the surface with the proposed underground facility. The effect was to reify the repository, not only through the simplification of the model but also through its imaginative projection into the underground. By contrast, the geoscientific research that had led to the selection of the site seemed to be increasingly relegated to background information. For example, the three-dimensional model barely illuminated the Opalinus clay stratum, which only appeared visually as a bounded layer on the shadowed back of the model. This new display revealed that the subterranean stratum, so meticulously staged prior to site selection, was no longer in the spotlight. Instead, it revealed that the subterranean had morphed into a nuclear stratum—as it now entangled an imagined postnuclear future in which nuclear waste was safely contained within the Opalinus clay. While the stability of the host rock was taken for granted, the focus had shifted to the future, which, as Keating and Storm (2023) point out, seems much more speculative in nature.

## Conclusion

This paper has examined how Nagra's communication experts unearthed the stratified subterranean of northern Switzerland to the attention of the public during the recent drilling campaign. They did this, as illustrated, by arranging and staging a series of political materials not only as visual representations but also as tangible evidence of the existence of a subterranean clay stratum in the area. This included a series of scientific artifacts such as lithographic profiles, rock samples, and drill cores that were “cut off from their original context” (van Geert, 2019: 135) and integrated into the narrative and institutional context of science communication. It also included a series of artifacts deliberately produced for the purposes of science communication, information, and illustration. These artifacts were performed in successive events over the course of the campaign and served as epistemic and aesthetic devices for subterranean sensing. Maps, models, and materials were strategically assembled to objectify and aestheticize the subterranean so that the public could not only learn or know about the Opalinus clay host rock but also experience it. It reflects a broader shift in Nagra's approach to science communication in the course of the campaign moving away from the dissemination of information toward the management of emotions.

Against this background, this paper has provided a fine-grained analysis of the strategic reimagining of the subterranean that has conditioned the recent selection of a site for a deep geological repository in Switzerland. It shows that Nagra's science communication experts presented the Opalinus clay to the public as a stable stratum in which the disposal project appeared straightforward and sound. In fact, this presentation was not the result of a predefined strategic communication plan. Rather, it was the result of tactical communications adjustments over the course of the drilling campaign—at times motivated by a desire to ensure transparency and trust, and at times driven forward in anticipation of controversy. The staging of the stratified subsurface was thus a gradual, improvised process that informed Opalinus clay as a political material and as a favorable host rock for nuclear waste disposal. Despite the diversity of practices and performances involved, it has allowed Nagra to forge and entangle a geosocial imaginary around clays competence to solve Switzerland's nuclear waste problem in the long term. The enactment of clay discussed in this paper thus provides a valuable example of what Orsini (2024) has called the “myth of containment,” evoked in the nuclear Anthropocene to manage the vibrancy of radioactivity and radioactive waste.

The project of deep geological disposal has certainly made the subterranean in northern Switzerland a site for political projections and imaginings of a (post-)nuclear future (see also Melo Zurita et al. 2017). Furthermore, it has opened up the subterranean as an epistemic, calculable, or governable space for the advancement of the disposal project (see also Braun, 2000; Elden, 2013). More fundamentally, however, this paper has shown how a clay stratum became enrolled into a political process at the surface. The stabilization of clay has helped to render deep geological disposal feasible not only in a technoscientific sense but above all in a sociopolitical sense. In this way, Opalinus clay has *come to matter*—both literary and politically—within Switzerland's contentious nuclear (waste) history. Li (2013: 400) has similarly drawn on the metaphor in her research on aquifers, using the notion of *matter* to capture the knowledge controversies through which “elements of the landscape acquire political significance and become the focus of public concern.” The ontopolitical struggle discussed in this paper, however, is much more one-sided: to (mis-)use Latour's (2004) vocabulary, it is less about making the subterranean a *matter of concern* than a *matter of fact*. The performance of the stratified subterranean has been primarily a hegemonic project in Switzerland, depoliticizing the nuclear waste issue by geologizing the debate and naturalizing an account of a stable and stratified earth.

## Highlights

- The Swiss nuclear waste organization has framed clay as a stable host rock to render deep geological disposal feasible in the eyes of citizens.
- Science communication experts have enacted, or brought into being, a stable Opalinus clay stratum as an epistemic and governable object.
- The enactment of clay strata consolidates a geosocial formation in which the deep geological disposal project can advance as a technical and political project.
- In light of a contested nuclear (waste) history, geology is constitutive in postnuclear futures.

## Notes

1. Not considered in this paper are events that Nagra organized for more informed public stakeholder groups involved in nuclear waste governance through participatory processes (see SFOE, 2008).

2. Author's fieldnotes, 17 May 2019 (own translation).

3. Author's fieldnotes, 17 May 2019 (own translation).

4. Author's fieldnotes, 19 September 2022 (own translation).
